# Supplementary material for: Why link diverse citizen science surveys? Widespread arboreal habits of a terrestrial amphibian revealed by mammalian tree surveys in Britain
Source: PLoS One. 2022 Jul 6;17(7):e0265156. doi: 10.1371/journal.pone.0265156 (PMC9258833; doi:10.1371/journal.pone.0265156)
Supplement: S1 Table — (DOCX) [file pone.0265156.s001.docx]

| **Measure type** | **Model** | **npar** | **AIC** | **BIC** | **logLik** | **Deviance** | **ChiSq** | **Df** | **Pr(>Chisq)** |
| --- | --- | --- | --- | --- | --- | --- | --- | --- | --- |
| **Tree height (m)** | Model without toad variable | 3 | 8218.343389 | 8234.032905 | -4106.171695 | 8212.343 | NA | NA | NA |
| **Tree height (m)** | Model with toad variable | 4 | 8220.187905 | 8241.107261 | -4106.093953 | 8212.188 | 0.155484 | 1 | 0.693349 |
| **DBH (cm)** | Model without toad variable | 3 | 13022.92319 | 13038.59964 | -6508.461597 | 13016.92 | NA | NA | NA |
| **DBH (cm)** | Model with toad variable | 4 | 13024.65678 | 13045.5587 | -6508.328389 | 13016.66 | 0.266416 | 1 | 0.605746 |
| **PRF height (m)** | Model without toad variable | 4 | 7294.0685 | 7315.619337 | -3643.03425 | 7286.069 | NA | NA | NA |
| **PRF height (m)** | Model with toad variable | 5 | 7294.172352 | 7321.110898 | -3642.086176 | 7284.172 | 1.896149 | 1 | 0.16851 |
| **Entrance height (cm)** | Model without toad variable | 4 | 17442.09283 | 17463.53325 | -8717.046416 | 17434.09 | NA | NA | NA |
| **Entrance height (cm)** | Model with toad variable | 5 | 17444.08187 | 17470.88239 | -8717.040935 | 17434.08 | 0.010962 | 1 | 0.916613 |

Table S1 (Supplementary material). Log likelihood test for a general linear mixed model comparing a model with the term of interest (i.e., toads present), with model without the term of interest.
